# Toxicity of Insecticides and Miticides to Natural Enemies in Australian Grains: A Review

**DOI:** 10.3390/insects12020187

**Published:** 2021-02-22

**Authors:** Kathy Overton, Ary A. Hoffmann, Olivia L. Reynolds, Paul A. Umina

**Affiliations:** 1 Cesar Australia, 293 Royal Parade, Parkville, VIC 3052, Australia; oliviareynolds@susentom.com (O.L.R.); pumina@cesaraustralia.com (P.A.U.); 2 Pest and Environmental Adaptation Research Group, School of BioSciences, Bio21 Institute, The University of Melbourne, Parkville, VIC 3052, Australia; ary@unimelb.edu.au

**Keywords:** beneficial insect, broadacre, chemical, predator, parasitoid, pesticide

## Abstract

**Simple Summary:**

Controlling invertebrate pests in crop fields using chemicals has been the main management strategy within the Australian grains industry for decades. However, chemical use can have unintended effects on natural enemies, which can play a key role in suppressing and controlling pest outbreaks within crops. We undertook a literature review of studies that have conducted chemical toxicity testing against arthropod natural enemies relevant to the Australian grains industry to examine trends and highlight research gaps and priorities. Most toxicity trials have been conducted in the laboratory, with few at larger, and hence, industry-relevant scales. Researchers have used a variety of methods when conducting toxicity testing, making it difficult to compare within and across different species of natural enemies. Furthermore, we found many gaps in testing, leading to unknown toxicity effects for several key natural enemies, some of which are economically important predators and parasitoids. Through our review, we make several key recommendations for future areas of research that could arm farmers and their advisors with the knowledge they need to make informed decisions when it comes to controlling crop pests.

**Abstract:**

Continued prophylactic chemical control to reduce pest populations in Australian grain farming systems has limited the effectiveness of biological control via natural enemies in crops within an integrated pest management (IPM) framework. While a variety of data is available to infer potential non-target effects of chemicals on arthropod natural enemies, much of it may be irrelevant or difficult to access. Here, we synthesise the literature relevant to Australian grain crops and highlight current knowledge gaps for potential future investment. A range of testing methodologies have been utilised, often deviating from standardised International Organization for Biological Control (IOBC) protocols. Consistent with findings from over 30 years ago, research has continued to occur predominantly at laboratory scales and on natural enemy families that are easily reared or commercially available. There is a paucity of data for many generalist predators, in particular for spiders, hoverflies, and rove and carabid beetles. Furthermore, very few studies have tested the effects of seed treatments on natural enemies, presenting a significant gap given the widespread global use of neonicotinoid seed treatments. There is a need to validate results obtained under laboratory conditions at industry-relevant scales and also prioritise testing on several key natural enemy species we have identified, which should assist with the adoption of IPM practices and decrease the reliance on broad-spectrum chemicals.

## 1. Introduction

Globally, large-scale agricultural enterprises, particularly in western agriculture, rely predominantly upon prophylactic chemical control and host plant resistance/tolerance to reduce invertebrate pest populations [1,2]. This has resulted in an over-reliance on chemicals and the emergence and spread of pesticide resistance in a wide range of pest species across an array of agroecosystems [3,4]. The Australian grains industry is no exception, with the use of inexpensive broad-spectrum pesticides as the central tactic utilised for pest control [3,5,6]. Australian-grown grains (cereals, oilseeds, pulses) encompass a key agricultural commodity, which is forecasted to contribute approximately 25% (estimated AUD $16.39 billion out of AUD $65.09 billion) in production value across all Australian agricultural sectors in 2020–2021 [7]. However, mounting failures to manage key invertebrate pest species, including the diamondback moth (*Plutella xylostella* (Linnaeus)), redlegged earth mite (*Halotydeus destructor* (Tucker)), green peach aphid (*Myzus persicae* (Sulzer)), and corn earworm/cotton bollworm (*Helicoverpa armigera* (Hübner)) [3], threaten the growth, sustainability, and profitability of the industry. With a diminishing number of effective chemical control options, the Australian grains industry is left with limited cost-effective crop protection strategies for pest management [3]. 

The goal of Integrated Pest Management (IPM) is to shift from the traditional use of prophylactic pesticide treatments towards more sustainable farming approaches. One of the pillars of IPM is the utilisation and preservation of arthropod natural enemies (predators and parasitoids) in agroecosystems to biologically control pest species [8,9,10,11]. Strategies exist that permit farmers to manipulate and augment predator and parasitoid densities by releasing mass-reared commercially available species (augmentative biological control) [12,13] or to conserve existing populations through landscape diversification by including or conserving non-crop and resource-rich habitat (conservation biological control) [10,14,15]; however, the application of these strategies within a broadacre context can have varying success [9]. Sustainable management strategies such as the choice of selective chemicals that are less harmful or disruptive towards natural enemies further complement these systems. Entomopathogenic nematodes from the families Steinernematidae and Heterorhabditidae, and their mutualistically associated bacteria, can also contribute to the biological control of invertebrate pests within agricultural systems, suppressing a variety of economically significant pest species [16,17,18,19]. Furthermore, an array of microbial biological control agents, consisting of bacteria, bacteriophages, fungi, yeasts, and viruses, have been harnessed across agroecosystems globally to target a range of economically important diseases and pests [20].

In Australia, natural enemies and IPM are central to pest control in horticulture [9] and cotton [21] agroecosystems. However, the Australian grains industry has had a slower IPM adoption rate [6,22]. In part, this is thought to be the result of several perceived hurdles and shortcomings of IPM in a broadacre context, which include a lack of training in pest and natural enemy monitoring and identification, limited in-field studies on natural enemies and an understanding of their true impact on pest numbers, a lack of replicated demonstration trials, and low confidence in IPM [6,23,24]. Compared with cotton agroecosystems, the crops encompassed under the grains umbrella are incredibly diverse, with crops attacked by a multitude of pests that can vary in prevalence as well as across climactic and geographic zones [9]. Furthermore, arthropod natural enemy distribution and frequency can vary considerably across different growing regions and are highly dependent on factors such as seasonality, availability of alternative food sources to sustain populations, and the history and frequency of pesticide input within the agroecosystem. Additionally, as grain crops in Australia are typically grown in a variety of rotation plans over significantly larger areas compared with horticultural and cotton agroecosystems, the formulation of IPM strategies is further complicated [9]. 

There is a comprehensive range of insecticides and miticides currently registered across multiple chemical Modes of Action (MoA) groups (as classified by the Insecticide Resistance Action Committee, IRAC [25]) in Australian grains [26]. However, MoA groups 1 (1A: carbamates, 1B: organophosphates), 3A (pyrethroids), and 4A (neonicotinoids) remain the most utilised [3], often because they are significantly less expensive than other chemistries. Organophosphates and synthetic pyrethroids accounted for >85% of all pesticide use in Australian cereals and legumes from 2009 to 2016 [3] and are known for their harmful impacts on non-target populations of natural enemies [27,28]. Frequent chemical use can result in cascading shifts throughout trophic levels, which in turn can influence the structure of pest communities, such as through secondary pest outbreaks [1,29]. Furthermore, despite international momentum surrounding the ban of neonicotinoids in agriculture due to their impact on bee colonies, wild pollinators, and natural enemy abundance [30,31,32], neonicotinoids (particularly imidacloprid, thiamethoxam, and clothianidin) continue to be widely used in Australian grain crops; these are commonly applied as seed treatments prior to sowing as a means to reduce pest infestations and damage incurred at crop establishment and early plant growth stages [3,24].

An important step towards greater IPM adoption in Australian grain systems is to enhance the impact of natural enemies. In order to achieve this, we must understand the non-target effects of insecticides and miticides on those arthropod natural enemies that offer the greatest level of biological control. Non-target effects of insecticides and miticides are frequently investigated across an array of species, active ingredients, and crop-specific contexts, with a focus on both direct impacts (toxicity/mortality) and indirect or sublethal impacts (such as effects on fecundity, sex ratio, and emergence) [33]. Within an Australian grains context, several key studies have been conducted across multiple experimental scales, demonstrating the selectivity (or lack thereof) of different insecticides and miticides [34,35,36,37,38,39,40,41,42]. There is a huge diversity of specialist and generalist predatory invertebrates and parasitic Hymenoptera found in Australian grains [23,43,44,45]. Therefore, establishing the relevant research that has already been conducted and identifying potential future research priority areas, whether for specific MoAs or for natural enemy genera or families, are key to better understanding the potential non-target impacts of chemicals. While the integration of selective or “soft” chemicals into grain agroecosystems may alleviate some of the pressures placed on natural enemy populations, identifying the impacts that these active ingredients can have on each natural enemy species must first be understood. 

Here, we conducted a systematic quantitative review [46] of the literature to identify trends in experimental methodologies utilised by researchers when testing the toxicity of insecticides and miticides on genera of arthropod natural enemies that are important within Australian grains systems. Through the development of an extensive database, we gained insight into natural enemy taxa where toxicity testing is lacking (and should therefore be prioritised) and detected trends in the research conducted, specifically the chemical exposure routes and testing methodologies utilised. Finally, we show the number of observations found across a range of experimental scales and identify where laboratory findings have been validated in the field, and hence at industry-relevant scales. 

## 2. Materials and Methods

### 2.1. Determining Relevant Active Ingredients Used for Pest Management in Australian Grains

The InfoPest database [47] and the Australian Pesticides and Veterinary Medicines Authority (APVMA) Public Chemical Registration Information System Search (PubCRIS) database [48] were searched and a list of the current insecticide and miticide active ingredients registered for use in all grain crops was completed in October 2019. Amorphous silica was excluded from the total of 37 active ingredients identified as it is not industry-relevant across a broad scale. While imidacloprid and thiamethoxam are currently registered for use only as seed treatments in grains, and methiocarb is only registered for use as a pelleted bait for snails and slugs, we included all exposure methodologies for these three active ingredients during our systematic review. The active ingredients and their corresponding MoA group included in this study are listed in Table 1.

### 2.2. Arthropod Natural Enemy Species Included in the Systematic Review

The taxa (i.e., species, genus, or family) of arthropod natural enemies (henceforth referred to as natural enemies) relevant to Australian grain crops were derived from Holloway et al. [9]. To ensure we had thorough coverage of all important natural enemy taxa, we consulted with experts and researchers within the Australian grains industry. As a result, we used the taxa listed in the first column of Table 2 as search terms during the systematic review. Some of the species and genera we included in our review do not occur in Australia; however, due to a lack of data for related Australian species, we incorporated them as a source of comparison. Arthropod natural enemy names were taken as stated within the corresponding literature; however, synonyms for some species were also captured and subsequently adjusted to the current taxonomic classification, wherever appropriate (e.g., *Amblyseius* was changed to *Neoseiulus* where applicable).

### 2.3. Deriving Data from Publications, Databases, and Industry Reports

To identify all available published literature on the effects of active ingredients (Table 1) on natural enemies (Table 2) relevant to Australian grain crops, we searched the Web of Science and Google Scholar databases between October 2019 and January 2020. The search terms used were specific for each active ingredient and natural enemy taxon; i.e., we used the search term: “[natural enemy taxon] AND [active ingredient]”, e.g., “Aphidius AND pirimicarb”. If the search yielded no results, we used the broad natural enemy family, order, or common name until results were obtained: “[natural enemy taxon or common name] AND [active ingredient]”, e.g., “hoverfly AND cypermethrin”. Results were manually screened by title and abstract to identify articles that were relevant to our study. In conducting the review, we included ants, given they have been reported to predate on pests within crops [49,50]. However, as some species of ants can be crop pests, we only included data where ants were considered as a natural enemy in the context of the study and excluded data that testing against ants in a pest context.

In addition, we extracted data from grey literature sources: the International Organization for Biological Control (IOBC), the Biobest Group NV Side Effects Manual [51], Koppert BV [52], and the United States Environmental Protection Agency (EPA) ECOTOX [53] databases. For the EPA ECOTOX database, we searched for the effects of insecticides and miticides on natural enemies by searching for the species and active ingredient and then locating the original citation from which the data were derived. Furthermore, we searched the Pesticide and Beneficial Organisms reports produced by the IOBC-West Palaearctic Regional Section (WPRS) Working Group as additional sources of data. 

The IOBC mortality data was recorded as four different evaluation categories, ranging from 1–4 according to Sterk et al. [54]; therefore, explicit percentage mortality or percentage reduction in abundance after insecticide or miticide exposure was not always provided. Laboratory and extended laboratory studies were categorised as 1 = harmless (<30% mortality), 2 = slightly harmful (30–79% mortality), 3 = moderately harmful (80–99% mortality), and 4 = harmful (>99% mortality), and semi-field and field studies were categorised as 1 = harmless (< 25% mortality), 2 = slightly harmful (25–50% mortality), 3 = moderately harmful (50–75% mortality), 4 = harmful (>75% mortality). The Biobest and Koppert databases reported effects of active ingredients using the same categories as the IOBC for semi-field and field studies. 

To collect data from Australian industry reports (additional sources of grey literature), we searched “beneficial insect”, “beneficial invertebrate”, “natural enemies”, and “natural enemy” in the research section of the Grains Research and Development Corporation (GRDC; [55]), the Cotton Research and Development Corporation (CRDC; [56]), Hort Innovation [57], Wine Australia [58], and AUSVEG [59] websites between January 2020 and February 2020. All search results were scanned to ensure relevant toxicity studies on natural enemies were captured. 

### 2.4. Data Collected from Each Source

From publications, databases, and industry reports, we collected the following data, where available: (i) natural enemy species; (ii) natural enemy family; (iii) natural enemy order; (iv) active ingredient; (v) insecticide/miticide application rate (g a.i./ha, g a.i./L, g product/L, ppm, % a.i.); (vi) standardised field application rate in g a.i./ha (if applicable/available); (vii) mortality percentage, percentage reduction, range, or effect rating; (viii) time after treatment mortality was recorded (minutes, hours, days, weeks); (ix) pesticide exposure type (direct/topical contact, indirect contact, residual contact); (x) experimental scale (laboratory, extended laboratory, greenhouse trial, semi-field trial, or field trial); (xi) pesticide testing methodology; (xii) if the study was conducted in Australia or internationally; and (xii) data source.

As a broad range of testing procedures were recorded, we further categorised the methodologies used to expose insecticides and miticides to natural enemies as illustrated in Table 3. Exposure methodologies categorised as “spray application” encompass all studies where the insecticide or miticide tested was sprayed and thus could be converted to a standardised field rate. Methodologies categorised as “coated/treated substrate” encompass studies where the insecticide or miticide was applied without any sprays being conducted (e.g., plant leaves/leaf discs dipped into the chemical solution, the solution was pipetted onto soil or sand to test the effects on soil dwelling natural enemies, a vial or petri dish was coated with the chemical solution, or the solution was pipetted directly onto filter paper).

Stringent criteria were established to ensure the data included in our study was appropriate. To be included, studies must have measured the mortality of the natural enemy using either a before–after or control–treatment experimental design. Furthermore, studies that reported the mean number of natural enemies observed in before–after designs but did not provide a transformed percentage reduction were excluded. Data sources that were exempt from this were databases (IOBC, Koppert, Biobest), IOBC reports, and field studies where an IOBC rating was provided, as the reported effect ratings encapsulated percentage mortality. Studies that tested sublethal effects were not included in our review, as our aim was to identify the direct impacts of pesticide exposure/contact. As an individual study may have tested multiple active ingredients (and/or MoAs) as well as multiple natural enemy taxa or experimental scales, we incorporated each distinct test as a separate entry in the database. The full database is provided in the Supplementary Materials (see Table S1). 

### 2.5. Creating Figures

The aggregate function in R [60] was used to summarise our findings and establish the number of entries (OBS) observed across different families and orders of natural enemies relevant to the Australian grains industry across insecticide and miticide MoAs, experimental scales, testing methodologies, and exposure routes. Insecticides and miticides were categorised into their MoA groups in accordance with the IRAC [25] (Table 1). The package ggplot2 was used to create tile plots in R [61]. Prior to the construction of figures, we removed any duplicates within our dataset where we captured mortality within a particular treatment recorded at multiple timepoints (e.g., 24, 48 and 72 h after treatment) and at multiple lifecycle stages. Of the 2786 distinct entries identified, the lowest taxonomic classifications that were specified across the entire dataset were 86.00% for species (2396 entries), 8.22% for genus (229 entries), 3.37% for family (94 entries), and 2.40% for order (67 entries). Due to the large diversity of species found during our review (Table 1), we summarised our findings at the genus level. However, we also created figures at higher taxon levels (i.e., family and order) to further understand if observed trends at the genus level were upheld with increasing taxonomic rank. 

To compare the distribution of the lowest taxon reported for data generated internationally or in Australia, we created two datasets, finding contrasting distributions. Of the 2336 distinct entries conducted internationally (constituting 83.88% of the entire dataset created), the lowest taxonomic classifications were: 91.48% for species (2137 entries), 5.90% for genus (138 entries), 1.97% for family (46 entries), and 0.64% for order (15 entries). In contrast, of the 450 entries for toxicity data generated in Australia, the lowest classifications were: 57.56% for species (259 entries), 20.22% for genus (91 entries), 10.67% for family (48 entries) and 11.56% for order (52 entries). Given the discrepancy in the proportion of the lowest defined taxonomic classifications between international and Australian studies, we created figures to investigate differences in research efforts across various MoAs and natural enemy families, rather than species and genus, to ensure the figures were based on comparable proportions. 

## 3. Results

### 3.1. Number of Entries across Relevant MoAs, Genera, Families, and Orders

**Figure 1:**
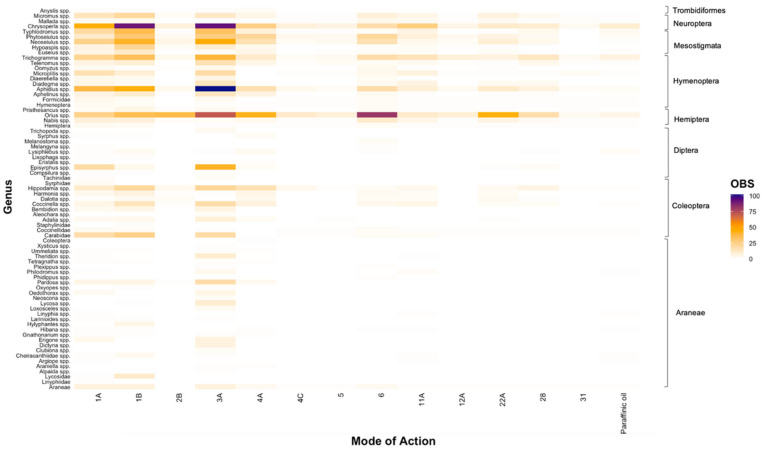
The number of entries (OBS) that tested the toxicity of chemical Modes of Action for natural enemy genera relevant to the Australian grains industry. Genera are grouped by order, which is illustrated on the right x-axis. Note: As some studies did not classify individuals tested to the genus level, some families (Carabidae, Coccinellidae, Formicidae, Linyphiidae, Lycosidae, Staphylinidae, Syrphidae, Tachinidae) and orders (Araneae, Coleoptera, Hemiptera, Hymenoptera) appear in the figure.

Of the 2786 unique entries conducted across relevant MoAs and natural enemy families relevant to Australian grain crops, we found the majority of entries were focused on older chemistries; most entries tested the toxicity of synthetic pyrethroids (Group 3A; 25.97%), organophosphates (Group 1B; 19.73%), and carbamates (Group 1A; 13.61%) (Figure 1). Considerably less research was focused on avermectins (Group 6; 8.99%), neonicotinoids (Group 4A; 8.20%), *Bt* (Group 11A; 5.09%), oxadiazines (Group 22A; 4.76%), diamides (Group 28; 3.37%), and phenylpyrazoles (Group 2B; 2.83%) (Figure 1). Furthermore, less than 2% of entries tested the toxicity of paraffinic oil, sulfoximines (Group 4C), spinosyns (Group 5), diafenthiuron (Group 12A), and NPV (Group 31) (Figure 1). These findings correlate somewhat with the number of cumulative studies involving each MoA undertaken between 1975 and 2019, as recorded in our database (Figure S1). Moving through time from the first published study for each MoA, there was a relatively consistent increase in the number of studies for several chemicals, particularly Groups 1A, 1B, 3A, 4A, 6, and 28. Conversely, there was no such trend for other MoAs, such as Groups 11A, 12A, 31, and paraffinic oil.

At the genus level, most toxicity testing was conducted on *Orius* spp. (14.29%), *Chrysoperla* spp. (12.86%), and *Aphidius* spp. (9.38%), constituting over a third (36.53%) of all entries identified in our review (Figure 1). Within the order Coleoptera, research on genera of ladybird beetles (*Hippodamia* spp., *Coccinella* spp., *Harmonia* spp.) predominated, with considerably less research conducted on carabid beetles and rove beetles (Carabidae, Staphylinidae, *Aleochara* spp., *Bembidion* spp., *Dalotia* spp.; Figure 1). Within the order Mesostigmata, research was conducted mainly across three genera of mites: *Neoseiulus* spp. (6.20%), *Phytoseiulus* spp. (5.05%), and *Typhlodromus* spp. (4.15%), with considerably less conducted on *Euseius* spp. (1.15%) and *Hypoaspis* spp. (1.76%; Figure 1). Furthermore, very little testing was conducted for genera within the orders Araneae, Diptera, and Trombidiformes (Figure 1). Within Diptera, most research was conducted on *Episyrphus* spp. (2.47%), although most of this testing was against synthetic pyrethroids (Group 3A; Figure 1). For Araneae, the lowest taxonomic level commonly reported by researchers was Order (Figure 1). 

**Figure 2:**
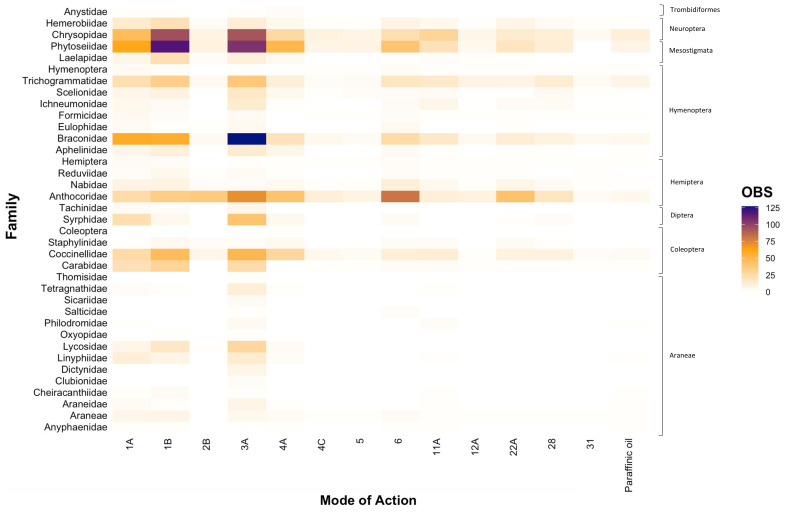
The number of entries (OBS) that tested the toxicity of chemical Modes of Action for each natural enemy family relevant to the Australian grains industry. Note: Araneae, Coleoptera, Hemiptera, and Hymenoptera are included as they were the lowest taxonomic classification provided in some studies.

At the family level, we similarly found that toxicity testing was dominated by research conducted across a limited number of groups, particularly Phytoseiidae (16.55%), Anthocoridae (14.29%), Chrysopidae (13.72%), Braconidae (12.57%), Coccinellidae (7.56%), and Trichogrammatidae (6.70%) (Figure 2). Consistent with our findings at the genus level (Figure 1), entries were scarce for various families within Araneae and Diptera, with each family typically comprising < 2% each of all entries. However, within the order Coleoptera, considerably less research was centred on the families Carabidae and Staphylinidae (Figure 2). Within the order Hymenoptera, research gaps were evident for the families Eulophidae and Formicidae (Figure 2). Furthermore, as observed at the genus level (Figure 1), the majority of toxicity testing was concentrated on carbamates (Group 1A), organophosphates (Group 1B), and synthetic pyrethroids (Group 3A), a finding that was consistent across all natural enemy families (Figure 2). 

**Figure 3:**
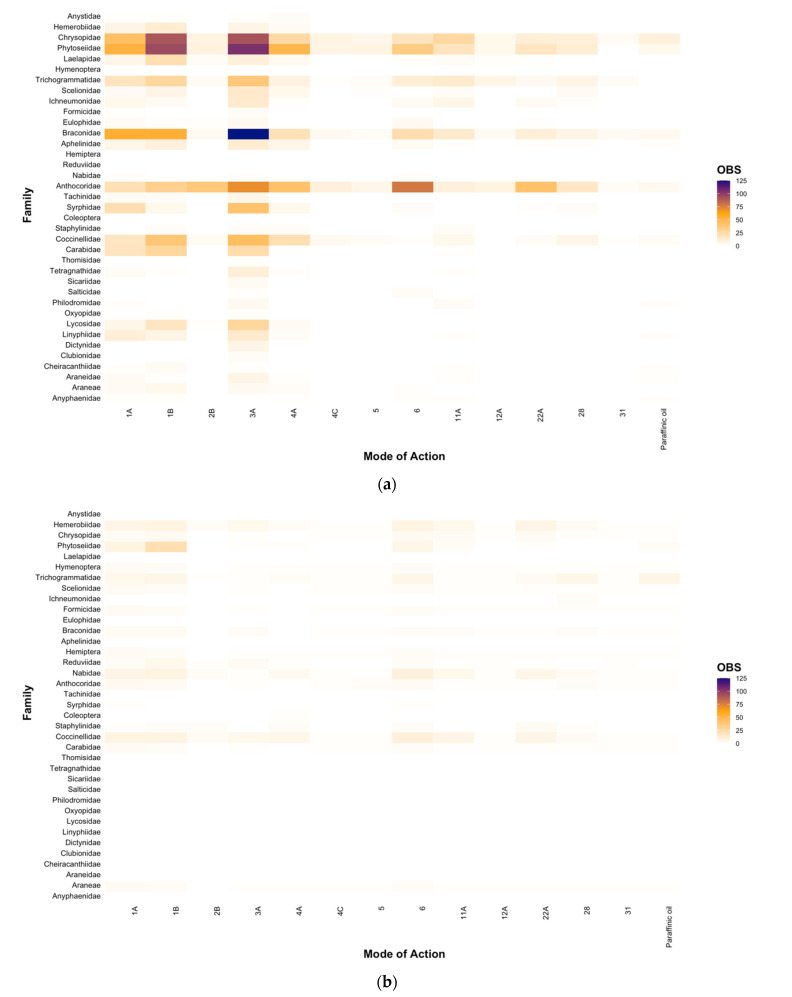
The number of entries (OBS) that tested the toxicity of chemical Modes of Action for each natural enemy family from studies conducted (**a**) internationally and (**b**) in Australia. Note: Araneae and Coleoptera are included as they were the lowest taxonomic classification provided in some studies.

The majority of insecticide and miticide toxicity research has been conducted internationally (83.88%; Figure 3A), with a paucity of research conducted in Australia (16.12%; Figure 3B). In particular, there has been little research effort investigating the toxicity effects on families of spiders in Australia (Figure 3B), with order (i.e., Araneae) typically being the lowest taxonomic classification reported. There were not any studies conducted for spiders across a range of families (i.e., Araneidae, Clubionidae, Dictynidae, Linyphiidae, Lycosidae, Oxyopidae, Philodromidae, Salticidae, Sicariidae, Tetragnathidae, Thomisidae). Furthermore, we did not find any studies that conducted toxicity testing for the families Anystidae, Aphelinidae, Eulophidae, Ichneumonidae, Laelapidae, Syrphidae, and Tachinidae, which encompass key predators and parasitoids within the Australian grains industry. Within Australia, most of the testing across MoAs was undertaken on the families Phytoseiidae, Coccinellidae, Nabidae, and Hemerobiidae (Figure 3B), however the data generated were still very limited relative to studies conducted internationally (Figure 3A). Consistent with our findings when looking at the distribution of data at a genus and family level (Figure 1 and Figure 2, respectively), data generated internationally focused mainly on broad-spectrum MoAs (i.e., Groups 1A, 1B, 3A), with fewer entries on newer and more selective chemistries (Figure 3A). 

**Figure 4:**
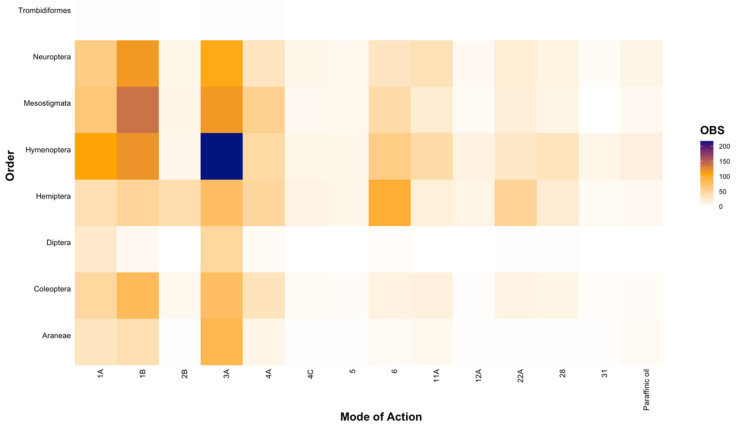
The number of entries (OBS) that tested the toxicity of chemical Modes of Action for each natural enemy order relevant to the Australian grains industry.

At a higher taxonomic level (i.e., Order), we found that trends observed at the family level were upheld (Figure 4), with most entries investigating the toxicity of broad-spectrum MoAs (Groups 1A, 1B, and 3A at 13.61%, 19.73%, and 25.97%, respectively; Figure 4), although we also found that considerable research was conducted on avermectins (Group 6; 8.99%) and *Bt* (Group 11A; 5.09%) (Figure 4). Furthermore, the majority of the research effort was focused on the orders Hymenoptera (25.79%), Mesostigmata (18.30%), Hemiptera (17.48%), and Neuroptera (16.65%), with significantly less research on Diptera (3.26%) and Araneae (6.88%; Figure 4). For the order Trombidiformes, we found only a single study testing the effects of five active ingredients (specifically carbaryl, phosmet, lambda-cyhalothrin, imidacloprid, and thiamethoxam; [62]), constituting a mere 0.18% of all findings (Figure 4) and presenting a substantial knowledge gap.

### 3.2. Testing Methodologies and Exposure Route

**Figure 5:**
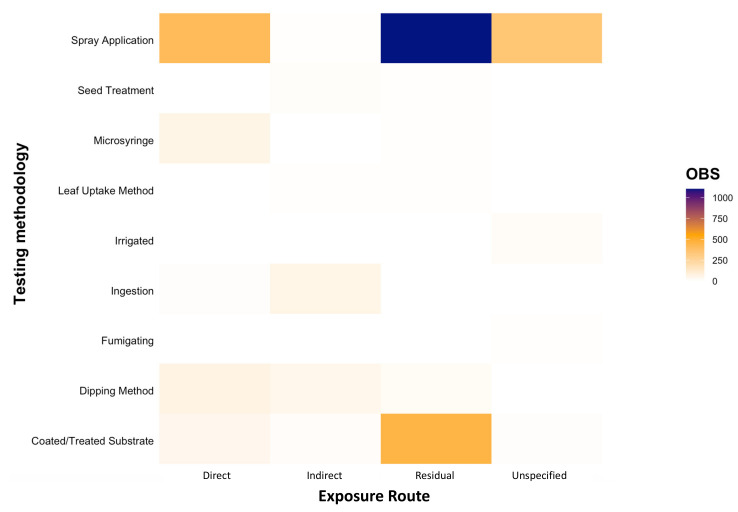
The number of entries (OBS) that used different methodologies and chemical exposure routes (direct, indirect, residual, or unspecified contact) when testing the toxicity of chemical Modes of Action against natural enemies relevant to the Australian grains industry.

We found a diversity of methodologies were utilised by researchers when testing the toxicity effects of insecticides and miticides on natural enemies (Figure 5). The majority of the research (39.71%) followed IOBC protocols, where a spray application was used to expose natural enemies to insecticides and miticides through dried residues (i.e., residual contact; Figure 5). However, 14.87% of entries tested the effect of direct spray contact on natural enemy mortality. Furthermore, 16.41% of entries tested residual contact exposure through a coated/treated substrate. Interestingly, despite the widespread use of seed treatments, both domestically and internationally, very few entries (0.61%) tested how natural enemies are affected when exposed through this pathway, highlighting a considerable gap (Figure 5). 

When looking more closely at the order level, we found toxicity effects for Araneae were tested predominantly via residual contact through coated/treated substrates and spray applications (Figure S2). For Coleoptera, most of the research was conducted through residual contact via spray applications, followed by direct contact via spray applications (Figure S3). Research testing toxicity against Diptera was conducted mostly via residual contact through coated/treated substrates, followed by residual contact via spray application (Figure S4). For Hemiptera, the majority of the research was conducted through residues applied through spray application (Figure S5). For Hymenoptera, most research was conducted through dried spray residues, followed by residual contact through coated/treated substrates (Figure S6). For Mesostigmata, most toxicity testing was generated through spray applications via unspecified, direct, and residual exposure methodologies (Figure S7), with residual contact studies also conducted through coated/treated substrate methodologies. Similarly, for Neuroptera, most research was performed using residual contact via spray application, although residual contact through coated/treated substrates was also widely used (Figure S8). For Trombidiformes, a single study tested the effects of insecticide and miticide exposure following IOBC protocols (Figure S9).

### 3.3. Experimental Scale

**Figure 6:**
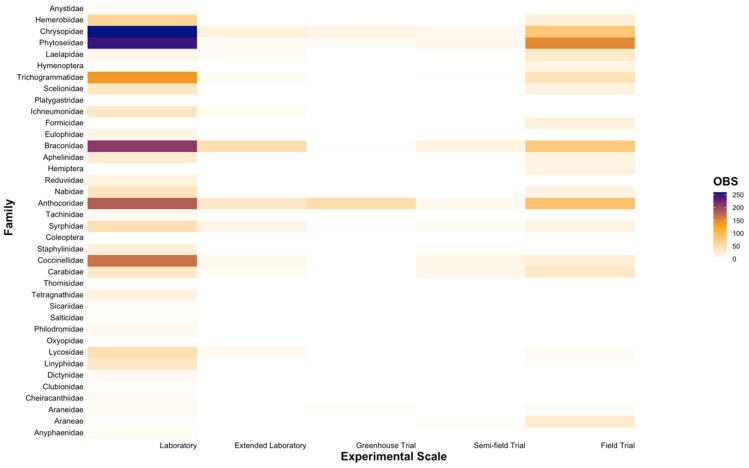
The number of entries (OBS) conducted for families of natural enemies across five experimental scales (Laboratory, Extended Laboratory, Greenhouse Trial, Semi-field Trial, Field Trial) across chemical Modes of Action relevant to the Australian grains industry.

**Figure 7:**
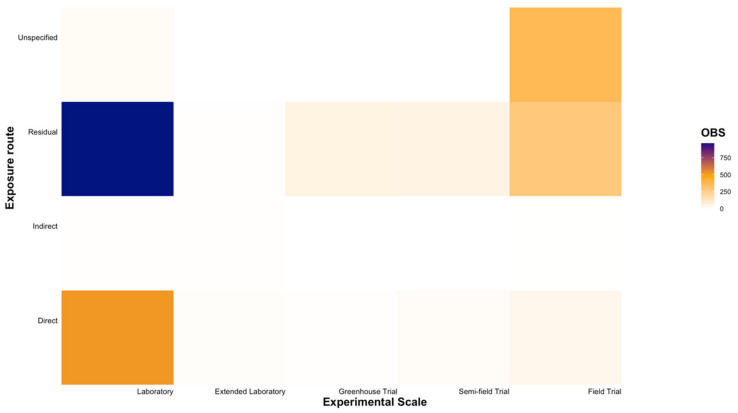
The number of entries (OBS) conducted across five experimental scales (Laboratory, Extended Laboratory, Greenhouse Trial, Semi-field Trial, Field Trial) using different insecticide and miticide exposure routes (Direct, Indirect, Residual, Unspecified) across chemical Modes of Action and natural enemies relevant to the Australian grains industry.

Of the five experimental scales, laboratory trials were most commonly utilised when testing the toxicity of insecticides and miticides against natural enemies (60.78% of all entries; Figure 6 and Figure 7). A distinct gap for various families within the order Araneae was observed at experimental scales beyond laboratory trials (Figure 6). While residual contact trials at the laboratory scale were the most commonly used approach for toxicity testing (34.49% of all entries), direct contact exposure at the same scale was also widely applied (18.59% of all entries; Figure 7). Field trials were commonly utilized (27.19% of all entries), with noticeably fewer entries conducted at extended laboratory, greenhouse, and semi-field scales (6.81%, 2.51%, and 2.76% of all entries, respectively; Figure 7).

## 4. Discussion

Insecticide and miticide toxicity effects testing on natural enemies relevant to the Australian grains industry have largely centred on broad-spectrum active ingredients and families of natural enemies that are commercially reared. Furthermore, testing methodologies were inconsistent across taxa of natural enemies, with experiments primarily conducted in laboratory settings. The bulk of the research effort has been performed internationally, with limited toxicity testing conducted within Australia. Spray applications appeared to be the most commonly used testing methodology when determining insecticide and miticide toxicity, although this varied among different natural enemy taxa. While residual contact seemed to be the main exposure route, direct contact was also a widely utilised approach; this deviates from IOBC standardised protocols and therefore raises the question of why this is the case. Set against the backdrop of our findings, we attempted to decipher how applicable and relevant internationally derived toxicity data are within an Australian context as well as how reliably we could infer laboratory findings at a field scale. 

### 4.1. Research Prioritisation Areas for Pesticides

The majority (59.31%) of research studies we identified focused on broad-spectrum chemistries (organophosphates, carbamates, and synthetic pyrethroids), which is congruent with insecticide and miticide usage in an Australian grains context and elsewhere around the world [3,63,64]. This is despite general knowledge of the consistently adverse effects of these groups of insecticides on natural enemies [65,66]. The large number of toxicity studies across these MoA groups may have stemmed from the fact that these chemistries have been used within an agricultural context for decades, due to the dozens of active ingredients and hence products available within each of these MoAs [25]. This view is supported somewhat by the number of cumulative studies undertaken on each MoA since 1975. For several MoAs, there has been a steady increase in studies since their introduction as pest control products in agriculture globally. However, other reasons are also likely to be important. For example, researchers may have conducted trials with specific chemicals to intentionally highlight their deleterious effects on particular natural enemies. 

There are considerable gaps for some chemical MoAs; in particular, there is a distinct lack of research on the effects of neonicotinoids when applied as a seed treatment. The importance of neonicotinoid seed treatments across grains crops in reducing pest outbreaks and virus damage associated with insect-vectors during crop establishment is well recognised [24,67]. Within Australia, imidacloprid and thiamethoxam are only registered for use as seed treatments, and between 2009 and 2016, neonicotinoids accounted for 11%, 3%, and 21% of chemical applications in cereals, legumes, and canola, respectively [3]. Neonicotinoid seed treatments are also frequently used across a broad range of agroecosystems globally [31,68,69]. Undertaking seed treatment studies can be challenging due to the difficulties associated with establishing the dynamics between the seed-treated host plant, the pest, and the natural enemy across trophic levels. However, given their widespread use in global agriculture, understanding both the immediate and long-term impacts of these chemicals on natural enemy communities warrants further exploration. 

We also found distinct knowledge gaps for some MoAs marketed as selective chemicals, with considerably less research on Groups 5 (spinetoram), 11A (*Bt*), 12A (diafenthiuron), 22A (indoxacarb), 28 (chlorantraniliprole), 31 (NPV), and paraffinic oils relative to broad-spectrum chemicals. Given the limited number of unique combinations for a given pest and crop combination [3,63], coupled with the recognition that natural enemies are essential for long-term pest management programs [15,22,70], readily accessible evidence is needed to understand the toxicity of chemical products so that farmers can make informed decisions about potential non-target effects [71]. The introduction of selective insecticides and miticides is believed to have shifted the motivation of toxicological research from studying the harm products can have on invertebrate pests to studying their harmlessness to natural enemies [70,72]. Given that selective products are generally more expensive compared with broad-spectrum chemicals, research demonstrating their selectivity against natural enemies should be conducted to justify their cost [73]. This therefore highlights a crucial need for cost-benefit analyses to be conducted across multiple grain crops to compare conventional “high-input” preventative and prophylactic spray tactics with the use of selective chemistries that promote natural enemy persistence within crops. For example, Macfadyen et al. [5] demonstrated that while insecticide use reduced pest abundance across five sites in southern Australia in both canola and wheat fields, this was generally not associated with higher yields. Furthermore, while feeding damage incurred by pests was observed in low insecticide input plots, this did not translate into yield losses [5], highlighting the importance of management thresholds within IPM frameworks. 

Although not covered in our review, there are numerous studies that have tested the effects of fungicides and herbicides on natural enemies (e.g., [35,36,74]). Though not designed to kill insects and mites, some fungicides and herbicides can be quite toxic to a range of invertebrates, including some natural enemies. For example, Bernard et al. [36] tested the direct and sub-lethal effects of 23 fungicides and one herbicide on *Euseius victoriensis* (Womersley) and reported varying toxicity effects. In particular, benomyl, carbendazim, mancozeb, wettable sulfur, and pyrimethanil were all considered highly toxic 48 h after exposure [36]. Therefore, understanding the toxicity effects of these pesticides warrants the same depth of investigation that is applied to insecticides and miticides, as their use will undoubtedly influence natural enemy communities.

### 4.2. Research Prioritisation Areas for Natural Enemies

As reported by Theiling and Croft [65] over 30 years ago, when it comes to toxicity testing on natural enemies, we found that Chrysopidae, Phytoseiidae, Anthocoridae, Braconidae, Coccinellidae, and Trichogrammatidae were the most commonly researched families [65]. We also found that toxicity data were heavily skewed towards species and families that are commercially reared; this is at least partly a result of the accessibility of particular natural enemies used in bioassays, influencing the amount of research conducted, rather than these natural enemies being the most “field-relevant” [65]. As identified in Thomson and Hoffmann [73], we also found IOBC standardised tests are typically restricted to relatively few species that either can be easily and economically cultured in the laboratory or are commercially reared. This in turn has led to an overwhelming amount of research effort concentrated on a select few species, which does not necessarily reflect the potential effects on the diverse genera of natural enemies encountered in the field, particularly from an Australian grains perspective [73]. 

Very few studies have tested the toxicity of insecticides and miticides on ants (Formicidae), hoverflies (Syrphidae), various families of spiders (Araneae), as well as rove and carabid beetles (Staphylinidae and Carabidae, respectively), all of which contribute significantly to pest control both in Australia and elsewhere. In particular, spiders are one of the most abundant predators recorded in grains crops in Australia, with spider assemblages able to effectively control many invertebrate pest populations [45]. The majority of studies on spiders have been conducted internationally, with the only Australian-generated data available through the Cotton Pest Management Guide [75], where toxicity effects are specified only to the order level (i.e., Araneae), and the underlying supporting data is not published. Globally, the importance of spiders has also been highlighted [76,77], and they are recognised as dominant epigeal arthropod predators that can control populations of a range of herbivorous arthropod pests in many agroecosystems [78]. Furthermore, Schmidt et al. [79] reported increases of 18%, 70%, and 172% in aphid populations when ground-dwelling generalist predators (spiders, carabid and staphylinid beetles), flying predators (predominantly syrphids, coccinellid beetles, gall midges), and a combination of the two groups, were experimentally reduced, respectively. This further highlights the lack of toxicity testing surrounding syrphids, spiders, and carabid and staphylinid beetles and the immediate need for research to fill these knowledge gaps. We also found a distinct lack of research within the family Anystidae (order: Trombidiformes), one of the key families of predatory mites that controls one of Australia’s major grains pest *H. destructor* [80], highlighting yet another important knowledge gap. In Western Australia, the predatory mite *Anystis wallacei* Otto has been found to kill 16,000 *H. destructor* in one pest generation when released at a density of 100/m^2^ in pasture plots [81]. 

Insect and mite orders that appear to have had a wealth of research conducted is overinflated by natural enemy species that do not occur in Australia. Most of the toxicity data within the orders Neuroptera and Hemiptera have been generated for *Chrysoperla* spp. and *Orius* spp., respectively, neither of which have much relevance in Australian grain crops. *Chrysoperla* spp. has undergone extensive toxicity screening, probably due to its worldwide distribution [82]; however, there is only one known species that occurs in Australia (i.e., *Chrysoperla congrua* (Walker)), and relative to the indigenous lacewing species *Mallada signatus* (Schneider) and *Micromus tasmaniae* (Walker), it is not as widely found nor recognised as an economically important natural enemy [83]. Similarly, the majority of toxicity data generated for Hemiptera is dominated by research conducted on *Orius* spp., which compared with other predatory bug species, such as the assassin bug (*Pristhesancus plagipennis* (Walker)) and damsel bug (*Nabis kinbergii* Reuter), is not considered a key natural enemy in Australian grains [9]. Few studies (*P. plagipennis*: [41,42]; *N. kinbergii*: [37]) have quantified the non-target impacts of insecticides and miticides for these two species, and significant research gaps still exist for the majority of the MoAs.

### 4.3. Research Methodologies and Limitations to Standardised Approaches

Despite a standardised methodology and protocol developed and published by the IOBC [84], studies continue to deviate from these methodologies, which raises questions not only about the practicalities of the criteria outlined by the IOBC, but also about whether the defined criteria align with the biology of tested natural enemies. IOBC protocols list “adequate ventilation” as a requirement [84], yet no specifications with regard to preventing desiccation from bioassay arenas are provided as a result of providing ventilation. In addition, protocols state that an “adequate exposure period before evaluation” in initial toxicity tests is required, yet little detail is provided as to what exposure periods are adequate [84]. Although researchers have generally used 48 and 72 h after treatment as experimental endpoints for parasitoids and predators, respectively, the lack of clarity has likely contributed to multiple experimental endpoints reported for the same genus. Some studies utilise 8 and 24 h after treatment as experimental endpoints, while others go beyond 48 or 72 h after treatment, which occasionally can reveal a delayed toxicity response towards certain active ingredients that may not have been captured if earlier endpoints were used. Collectively, these factors likely contribute towards the assortment of methodologies utilised by researchers, but they do not fully account for why different exposure routes are routinely tested. 

Natural enemies in the field can be exposed to pesticides directly through contact with spray droplets, residually through contact with contaminated surfaces, or orally (i.e., indirectly) through ingestion of contaminated food sources [70,85]. With these three routes of exposure, our finding of frequent deviations from IOBC conventions to test both direct and indirect contact with insecticides and miticides are therefore unsurprising. While findings from all three exposure routes hold relevance and applicability to industry, the toxicity effects reported cannot be directly compared across exposure routes, although they can still be used to bolster the selectivity or toxicity of a particular active ingredient. 

A significant advantage of IOBC testing protocols is that results can be compared across laboratories and agricultural systems [73]. However, using a standardised “representative” organism limits the toxicity data generated, as it does not consider the specific context in which chemicals are applied, the natural enemies present within this context, the cumulative effects of multiple chemical applications across a season, and the persistence of some insecticides and miticides within the soil over multiple seasons [38,73,86]. Furthermore, IOBC methodologies do not incorporate population effects, habitat complexity, and interactions with other natural enemies that may not be affected by the application of a particular chemical, which can assist in controlling a pest population [73]. At the community level, there is little information on pesticide effects [87,88], with research typically focused on secondary pest outbreaks that can occur after dominant natural enemies have been killed through chemical use [89,90]. The exposure to a particular active ingredient is unlikely to be similar between species, with some species exposed to residues more than others depending on their patterns of activity and location [70,71,91]. Therefore, only testing mortality and sublethal effects, while useful, is not necessarily the “best practice” in establishing the effects of pesticides on natural enemies. While valuable in generating initial data, they should be treated as a foundation for future research, after which population and community-level effects should be explored.

Although not captured in the figures presented above, a recurring issue we identified was studies failing to provide a standardised field rate (in g or mL of active ingredient/ha) or enough information to allow the field rate to be calculated (e.g., the industry equivalent spray rate in L/ha, the concentration of active ingredient of the product tested). This lack of information limits the ability of other researchers to compare previously tested active ingredients and reported toxicities for a particular genus (or species) of natural enemy. This issue, combined with researchers frequently deviating from standardised methodologies, handicaps our ability to make generalisations or draw conclusions about the toxicity of some active ingredients across various taxonomic classifications; therefore, while generalisations can be made, they should be made with caution.

### 4.4. Disconnect between Experimental Scales

While laboratory studies are valuable in establishing baseline toxicities, the effect that an insecticide or miticide will have on a natural enemy species in the field is a much more complicated and multidimensional process [71]. IOBC protocols recommend a tiered testing procedure, where a pesticide is tested in the laboratory first [92]. If harmful effects are not observed, the pesticide is considered compatible within IPM programs. However, if the pesticide is found to be toxic in laboratory settings, tests are performed in extended laboratory and/or semi-field trials to validate the effects observed [92]. We found that most research has been conducted at the laboratory scale, with research efforts also invested (albeit to a lesser degree) at field scales; extended laboratory, greenhouse trials, and semi-field trials are frequently skipped over. Our findings are consistent with previous reviews. For example, Croft [93] reported that much of the data surrounding the effects of pesticides and other toxicants on insects and other arthropods have been derived through laboratory studies, with contact tests as the preferred method of assessing pesticide impacts on natural enemies. Additionally, the SELCTV database found that less than a third of records in the compiled literature on the effects of pesticides on non-target species were conducted through field studies [65,94,95]. 

The consistency of insecticide and miticide toxicity across different experimental scales appears to be a complex issue (see: [95,96]), with contrasting findings reported. For example, Jenkins et al. [38] found that fields sprayed with broad-spectrum insecticides and miticides resulted in few treatment effects on non-target natural enemies; where detrimental effects on natural enemy populations were detected, effects were inconsistent both between natural enemy groups and field trials. However, Thomson and Hoffmann [97] validated IOBC toxicity ratings generated in the laboratory at the field scale for a range of natural enemy species and found that ratings generated at both spectra of experimental scales were correlated. Clearly, care should be taken when extrapolating laboratory scale assessments to predict potential impacts under field conditions, with differences likely arising under different crop types and environmental/growing conditions. 

### 4.5. Application of Findings to the Australian Grains Industry 

The majority of toxicity data has been generated internationally, and more specifically, in Europe. Acknowledging the key differences between farm management practices, the size (and scale) of farms/fields, and approved chemical products for use between European and Australian grain systems is crucial when considering the applicability of internationally generated findings within an Australian context. Furthermore, interspecific and intraspecific differences are likely to exist between Australian and international populations of natural enemies, with additional complications such as varying environments, climate, humidity, temperature, UV exposure (and therefore UV degradation), and rainfall patterns all potentially influencing the toxicity of chemicals. 

Until research is undertaken using Australian populations of natural enemies, researchers are left with little option but to infer potential toxicities from international work, using data generated either within the same species or within the same genus. This could be an issue, particularly for predatory mites, where mortality through pesticide contact is known to not only vary within a genus, but also within a species [98]. For example, direct contact laboratory assays testing the toxicity of abamectin on *Phytoseiulus persimilis* Athias-Henriot at 11.9 g ai/ha was reported to cause 100% mortality in adult females by Bostanian and Akalach [99], but Fiedler and Sosnowska [100] reported mortalities of 12% and 18% for adults exposed to higher rates of 28.3 and 45.3 g ai/ha, respectively. Ideally, toxicity data generated in Australia should be first performed at a laboratory scale, before being further validated in replicated semi-field and field trials encompassing multiple locations and hence different populations of natural enemies. However, the complexities of multiple crops nested under the grains umbrella poses challenges in terms of a “one size fits all” approach. Furthermore, registered rates for the control of pests varies between grain crops, between pest species, as well as occasionally between different Australian states and territories. These factors combined with different climactic conditions and geographic areas would amount to dozens of potential contexts that would need to be explored to identify the non-target effects for a single active ingredient. 

## 5. Conclusions

Escalating resistance issues [3], increased competition in the international market surrounding food safety concerns and pesticide residue limits [24], as well as greater attention now given to the impacts of insecticides and miticides on ecosystems and human health has meant the Australian grains industry is at an impasse. To meet demand and ensure profitability, the industry must maintain high production outputs while simultaneously adapting to more sustainable farming approaches. This impasse previously occurred in the Australian cotton industry, where the increased incidence of chemical control failures drove the widespread adoption of IPM [21,24]. For both conventional and *Bt*-cotton, farmers that used selective chemical control options in conjunction with IPM practices had equivalent or higher gross margins compared with farmers that continued to use conventional broad-spectrum insecticides and miticides; this was mainly attributed to the increased number of natural enemies present within IPM crops [21]. With an increasing number of selective products available for pest management, now is the time to incorporate IPM practices into grains agroecosystems. However, research must first identify which natural enemy species these chemicals are “softer” on and species where a toxic effect is observed. Our review illustrates that, while there have been numerous studies on the toxicity of insecticides and miticides on natural enemies relevant to the grains industry, there are many gaps relevant to local natural enemies.

## Figures and Tables

**Table 1:** Insecticide and miticide active ingredients currently registered for use within Australian grains (excluding amorphous silica) and their corresponding Insecticide Resistance Action Committee (IRAC) chemical Mode of Action (MoA) [25] groups.

IRAC Chemical MoA Group	Active Ingredients
1A	Carbaryl, methiocarb, methomyl, pirimicarb, thiodicarb
1B	Chlorpyrifos, diazinon, dimethoate, malathion, methidathion, omethoate, phosmet, trichlorfon
2B	Fipronil
3A	Alpha-cypermethrin, beta-cypermethrin, bifenthrin, cypermethrin, deltamethrin, esfenvalerate, gamma-cyhalothrin, lambda-cyhalothrin, permethrin, zeta-cypermethrin
4A	Imidacloprid, thiamethoxam
4C	Sulfoxaflor
5	Spinetoram
6	Abamectin, emamectin benzoate
11A	*Bacillus thuringiensis* (*Bt*)
12A	Diafenthiuron
22A	Indoxacarb
28	Chlorantraniliprole
31	Nuclear polyhedrosis virus (NPV)
-	Paraffinic oil

**Table 2:** Taxa included in our systematic review and their corresponding order and family. The findings for the taxon (species, genus, family, or order) used in the search term (i.e., in the first column) are presented in the last two columns, separating those where studies were conducted in Australia or internationally. A dash (i.e., -) in either of the last two columns indicates no relevant toxicity data were found.

Taxon used for Literature Search	Order	Family	Taxon Derived from International Studies	Taxon Derived from Australian Studies
Araneae	Araneae	Multiple families	*Alpaida venilia*, *Araniella cucurbitina*, *Araniella opisthographa*, *Argiope argentata*, *Cheiracanthium* sp., *Clubiona neglecta*, *Clubiona pallidula*, *Dictyna* sp., *Dictyna uncinata*, *Erigone atra*, *Gnathonarium exsiccatum*, *Hibana velox*, *Hylyphantes graminicola*, *Larinioides sclopetarius*, *Linyphia triangularis*, Linyphiidae, *Oedothorax apicatus*, *Ummeliata insecticeps*, *Lycosa pseudoannulata*, *Lycosa terrestris*, Lycosidae, *Neoscona theisi*, *Pardosa agrestis*, *Pardosa palustris*, *Pardosa prativaga*, *Pardosa pseudoannulata*, *Pardosa* spp., *Pardosa sumatrana*, *Oxyopes salticus*, *Philodromus aureolus*, *Philodromus cespitum*, *Phidippus audax*, *Plexippus paykulli*, *Loxosceles intermedia*, *Tetragnatha maxillosa*, *Theridion impressum*, *Xysticus cristatus*	Araneae
Carabidae	Coleoptera	Carabidae	*Agonum dorsale*, *Bembidion lampros*, *Bembidion obscurellum*, *Bembidion quadrimaculatum*, *Bembidion obtusum*, *Bembidion rapidum*, *Bembidion* spp., Carabidae, *Demetrias atricapillus*, *Harpalus pensylvanicus*, *Harpalus rufipes*, *Nebria brevicollis*, *Poecilus cupreus*, *Pterostichus chalcites*, *Pterostichus madidus*, *Pterostichus melanarius*, *Pterostichus melas italicus*, *Trechus quadristriatus*	Carabidae
*Catadromus lacordairei*	Coleoptera	Carabidae	-	-
*Notonomus gravis*	Coleoptera	Carabidae	*-*	*-*
*Adalia*	Coleoptera	Coccinellidae	*Adalia bipunctata*	-
*Coccinella*	Coleoptera	Coccinellidae	*Coccinella septempunctata, Coccinella* spp.	*Coccinella transversalis*
*Harmonia*	Coleoptera	Coccinellidae	*Harmonia axyridis*, *Harmonia* spp.	*Harmonia conformis*
*Hippodamia*	Coleoptera	Coccinellidae	*Hippodamia convergens*, *Hippodamia* spp., *Hippodamia variegata*	*Hippodamia variegata*
*Micraspis*	Coleoptera	Coccinellidae	-	-
*Dalotia*	Coleoptera	Staphylinidae	*Dalotia coriaria*	*Dalotia coriaria, Dalotia* sp.
Staphylinidae	Coleoptera	Staphylinidae	*Aleochara bilineata*, *Paederus* sp., Staphylinidae	-
*Melangyna viridiceps*	Diptera	Syrphidae	-	*Melangyna viridiceps*
*Simosyrphus grandicornis*	Diptera	Syrphidae	-	-
*Sphaerophoria macrogaster*	Diptera	Syrphidae	-	-
Syrphidae/Hoverfly	Diptera	Syrphidae	*Episyrphus balteatus*, *Eristalis tenax*, *Melanostoma fasciatum*, Syrphid, *Syrphus corollae*,	-
Tachinidae	Diptera	Tachinidae	*Compsilura concinnata, Trichopoda pennipes, Lixophaga diatraeae,* Tachinidae	-
*Orius*	Hemiptera	Anthocoridae	*Orius albidipennis*, *Orius insidiosus*, *Orius laevigatus*, *Orius majusculus*, *Orius niger*, *Orius* sp.	*Orius armatus, Orius* sp.
*Nabis*	Hemiptera	Nabidae	Nabidae, *Nabis roseipennis*	*Nabis kinbergii*
*Oechalia schellenbergii*	Hemiptera	Pentatomidae	-	-
*Pristhesancus plagipennis*	Hemiptera	Reduviidae	-	*Pristhesancus plagipennis*
*Aphelinus*	Hymenoptera	Aphelinidae	*Aphelinus abdominalis*, *Aphelinus certus*, *Aphelinus gossypii*, *Aphelinus mali*, *Aphelinus semiflavus*	-
*Aphidius*	Hymenoptera	Braconidae	*Aphidius colemani*, *Aphidius ervi*, *Aphidius gifuensis*, *Aphidius matricariae*, *Aphidius rhopalosiphi*, *Aphidius smithi*, *Aphidius* sp., *Aphidius* spp.	-
*Diaeretiella*	Hymenoptera	Braconidae	*Diaeretiella rapae*	-
*Lysiphlebus*	Hymenoptera	Braconidae	*Lysiphlebus confusus*	-
*Microplitis*	Hymenoptera	Braconidae	*Microplitis croceipes*, *Microplitis demolitor*, *Microplitis mediator*	*Microplitis demolitor*, *Microplitis* sp.
*Oomyzus*	Hymenoptera	Eulophidae	*Oomyzus sokolowskii*	-
Ant	Hymenoptera	Formicidae	Formicidae	Ants
*Diadegma*	Hymenoptera	Ichneumonidae	*Diadegma insulare*, *Diadegma semiclausum*	*Diadegma semiclausum*
*Diadromus*	Hymenoptera	Ichneumonidae	-	-
*Ichneumon*	Hymenoptera	Ichneumonidae	-	-
*Netelia*	Hymenoptera	Ichneumonidae	-	-
*Telenomus*	Hymenoptera	Scelionidae	*Telenomus busseolae, Telenomus podisi, Telenomus remus*	*Telenomus* sp.
*Trichogramma*	Hymenoptera	Trichogrammatidae	*Trichogramma cacoeciae*, *Trichogramma dendrolimi*, *Trichogramma pretiosum*, *Trichogramma* sp.	*Trichogramma pretiosum*
*Hypoaspis*	Mesostigmata	Laelapidae	*Hypoaspis aculeifer, Hypoaspis* spp.	-
*Euseius*	Mesostigmata	Phytoseiidae	*Euseius gallicus*	*Euseius victoriensis*
*Neoseiulus*	Mesostigmata	Phytoseiidae	*Neoseiulus californicus*, *Neoseiulus cucumeris*, *Neoseiulus fallacis*, *Neoseiulus womersleyi*	-
*Phytoseiulus*	Mesostigmata	Phytoseiidae	*Phytoseiulus persimilis*	*Phytoseiulus persimilis*
*Typhlodromus*	Mesostigmata	Phytoseiidae	*Typhlodromus occidentalis*, *Typhlodromus pyri*	*Typhlodromus doreenae*
*Chrysoperla*	Neuroptera	Chrysopidae	*Chrysoperla carnea*, *Chrysoperla rufilabris*	-
*Mallada signatus*	Neuroptera	Chrysopidae	-	*Mallada signatus*, *Mallada* sp.
*Micromus tasmaniae*	Neuroptera	Hemerobiidae	*Micromus tasmaniae*	*Micromus tasmaniae*
*Anystis*	Trombidiformes	Anystidae	*Anystis baccarum*	-
*Odontoscirus*	Trombidiformes	Bdellidae	-	-
*Neomolgus capillatus*	Trombidiformes	Bdellidae	-	-
Trombidiidae	Trombidiformes	Trombidiidae	-	-

**Table 3:** Categories used to define the testing methodologies used to expose natural enemies to insecticides and miticides.

Category	Exposure Methodologies Included
Coated/treated substrate	Leaf dip, leaf disc method, treated substrate (e.g., soil), coated glass vial method, petri dish, filter paper
Spray application	Spray, spray chamber, foliar spray, Potter Tower spray, hand-held/knapsack sprayer, boom sprayer
Dipping method	Natural enemy exposed to chemical solution by being dipped (includes slide dipping)
Fumigation	Experimental site/location is fumigated
Ingestion	Treated prey ingested by natural enemy
Irrigation	Experimental site/location is irrigated with chemical solution
Leaf uptake method	Leaf cutting is placed in chemical solution and then exposed to the natural enemy
Micro-syringe	Micro-syringe is used to topically apply chemical solution onto the thorax of the natural enemy
Seed treatment	Natural enemy is exposed to both pest and seed-treated plant
Unspecified	Chemical exposure methodology was not provided

## Data Availability

The full dataset has been provided as a Supplementary Materials.

## Supplementary Materials

The following are available online at https://www.mdpi.com/2075-4450/12/2/187/s1, Table S1: The raw datafile developed through the systematic literature review and used in subsequent analysis (in the second tab titled ‘Dataset’). The first tab titled ‘Explanation’ provides a detailed explanation for each of the column headers as well as different categories that can be found within each header (if relevant/applicable). Figure S1: The cumulative number of studies involving relevant active ingredients within each chemical Mode of Action (MoA) [25] group from 1975 to 2020. Note: this figure is based on the active ingredient(s) tested within a study and does not factor in natural enemy taxon. Data sources where no information was provided as to when the toxicity testing was conducted (i.e., Cotton Pest Management Guide, Biobest Database, Koppert Database, IOBC Database) were excluded. Figure S2: The number of entries (OBS) conducted for each contact type (direct, indirect, residual, or unspecified contact) for different testing methodologies across all experimental scales for Araneae. Figure S3: The number of entries (OBS) conducted for each contact type (direct, indirect, residual, or unspecified contact) for different testing methodologies across all experimental scales for Coleoptera. Figure S4: The number of entries (OBS) conducted for each contact type (direct, indirect, residual, or unspecified contact) for different testing methodologies across all experimental scales for Diptera. Figure S5: The number of entries (OBS) conducted for each contact type (direct, indirect, residual, or unspecified contact) for different testing methodologies across all experimental scales for Hemiptera. Figure S6: The number of entries (OBS) conducted for each contact type (direct, indirect, residual, or unspecified contact) for different testing methodologies across all experimental scales for Hymenoptera. Figure S7: The number of entries (OBS) conducted for each contact type (direct, indirect, residual, or unspecified contact) for different testing methodologies across all experimental scales for Mesostigmata. Figure S8: The number of entries (OBS) conducted for each contact type (direct, indirect, residual, or unspecified contact) for different testing methodologies across all experimental scales for Neuroptera. Figure S9: The number of entries (OBS) conducted for each contact type (direct, indirect, residual, or unspecified contact) for different testing methodologies across all experimental scales for Trombidiformes.
