# Supplementary material for: Toxicity of Insecticides and Miticides to Natural Enemies in Australian Grains: A Review
Source: Insects. 2021 Feb 22;12(2):187. doi: 10.3390/insects12020187 (PMC7927080; doi:10.3390/insects12020187)
Supplement: Supplementary file 1 [file insects-12-00187-s001.zip › Supplementary Files/SupplementaryMaterials_Figures.docx]

Supplementary Materials


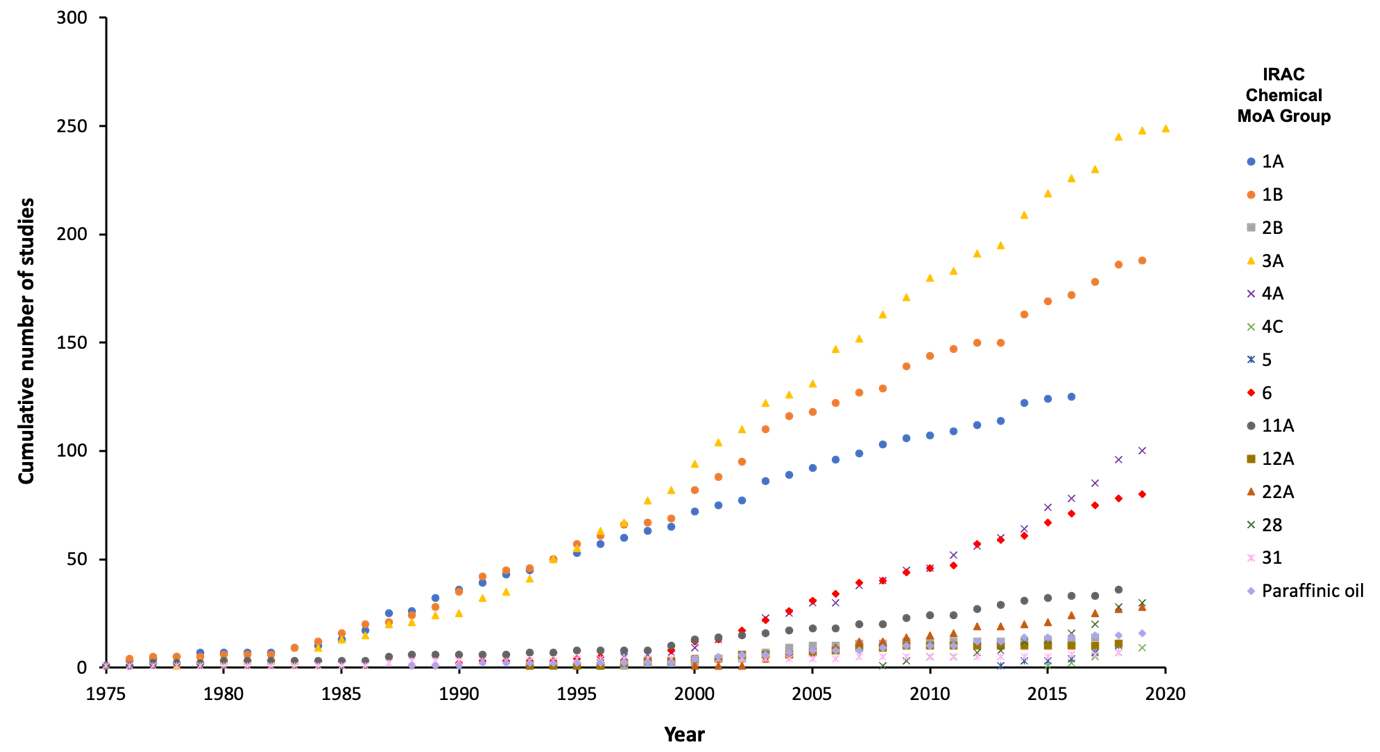


**Figure S1.** The cumulative number of studies involving relevant active ingredients within each chemical Mode of Action (MoA) [25] group from 1975 to 2020. Note: this figure is based on the active ingredient(s) tested within a study and does not factor in natural enemy taxon. Data sources where no information was provided as to when the toxicity testing was conducted (i.e. Cotton Pest Management Guide, Biobest Database, Koppert Database, IOBC Database) were excluded.

**Figure S2.** The number of entries (OBS) conducted for each contact type (direct, indirect, residual or unspecified contact) for different testing methodologies across all experimental scales for Araneae.

**Figure S3.** The number of entries (OBS) conducted for each contact type (direct, indirect, residual or unspecified contact) for different testing methodologies across all experimental scales for Coleoptera.

**Figure S4.** The number of entries (OBS) conducted for each contact type (direct, indirect, residual or unspecified contact) for different testing methodologies across all experimental scales for Diptera.

**Figure S5.** The number of entries (OBS) conducted for each contact type (direct, indirect, residual or unspecified contact) for different testing methodologies across all experimental scales for Hemiptera.

**Figure S6.** The number of entries (OBS) conducted for each contact type (direct, indirect, residual or unspecified contact) for different testing methodologies across all experimental scales for Hymenoptera.

**Figure S7.** The number of entries (OBS) conducted for each contact type (direct, indirect, residual or unspecified contact) for different testing methodologies across all experimental scales for Mesostigmata.

**Figure S8.** The number of entries (OBS) conducted for each contact type (direct, indirect, residual or unspecified contact) for different testing methodologies across all experimental scales for Neuroptera.

**Figure S9.** The number of entries (OBS) conducted for each contact type (direct, indirect, residual or unspecified contact) for different testing methodologies across all experimental scales for Trombidiformes.
